# Supplementary figures and images for: CRISPRi screens reveal genes modulating yeast growth in lignocellulose hydrolysate
Source: Biotechnol Biofuels. 2021 Feb 10;14:41. doi: 10.1186/s13068-021-01880-7 (PMC7874482; doi:10.1186/s13068-021-01880-7)

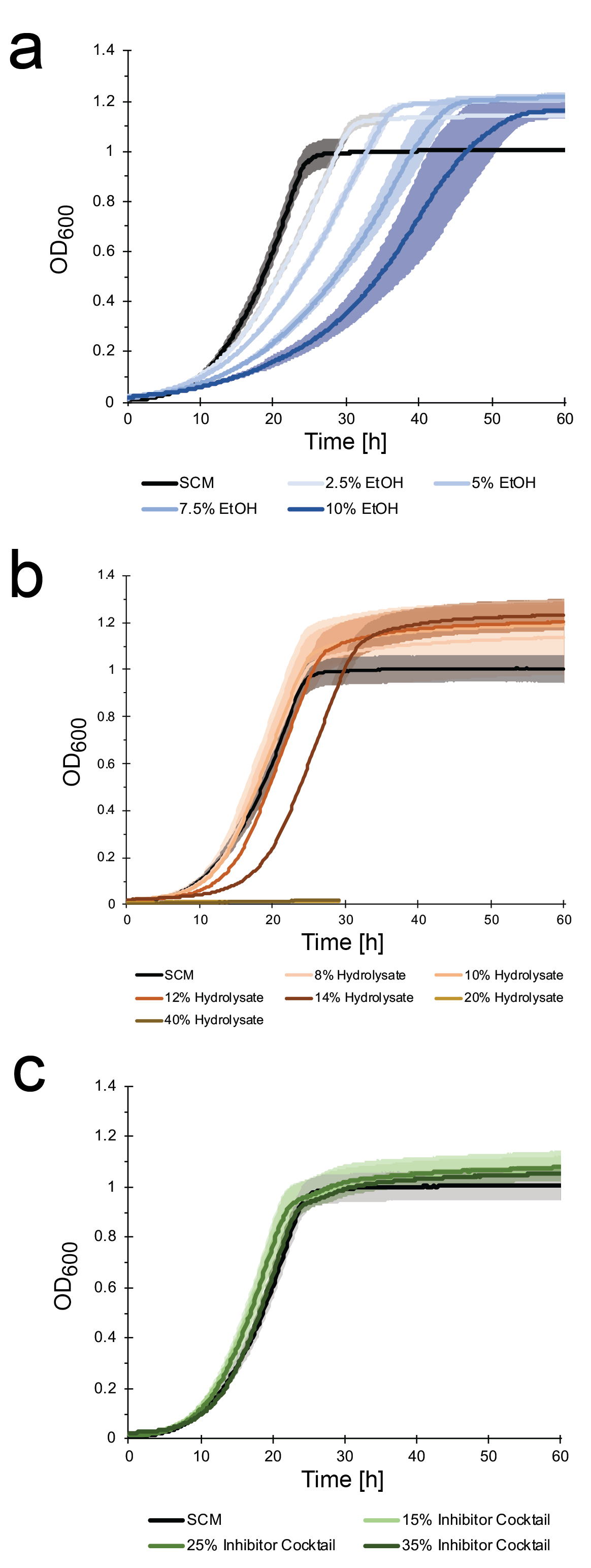

Supplement: Supplementary file 1 — Additional file 1: Figure S1. Yeast growth across media in 96-well plate format. The optical density at 600 nm (OD600, on y-axis) was quantified over time (x-axis) during growth of BY4743 in synthetic complete media with 2% glucose (SCM), as well as in SCM that was supplemented with different concentrations of (a) ethanol, (b) spruce hydrolysate or (c) inhibitor compound cocktail. The respective supplement concentrations are indicated individually. For the IC mixture, the 1x IC stock was diluted to the indicated percentages. The curves denote the average of n = 4 wells measured in 96-well format, normalized by subtraction of media background. [file 13068_2021_1880_MOESM1_ESM.png]

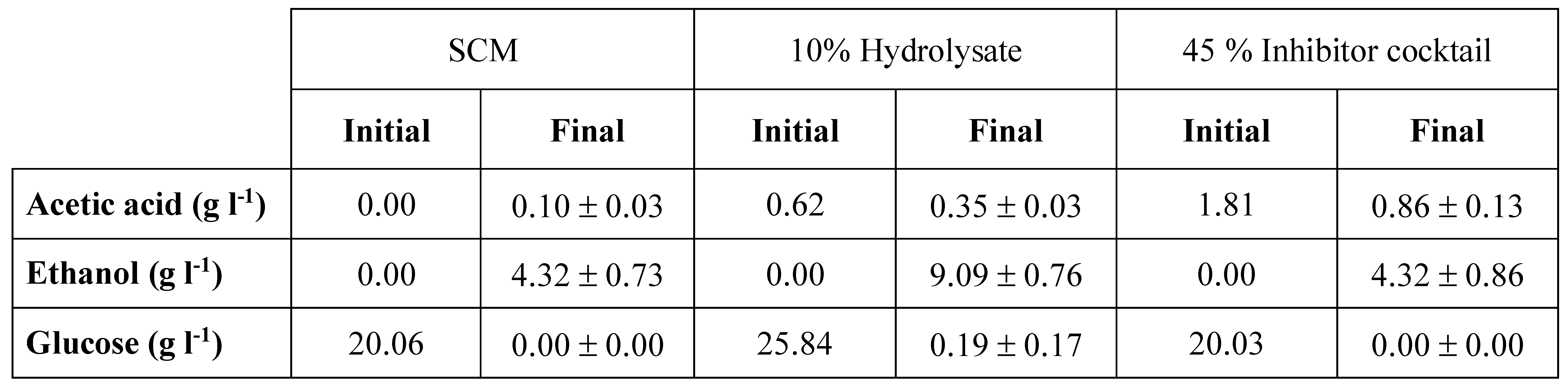

Supplement: Supplementary file 2 — Additional file 2: Table S1. HPLC measurements. Metabolites concentrations (g L−1), measured by HPLC, of yeast cultures grown in SCM, SCM+10% hydrolysate and SCM+45% inhibitor cocktail. Glucose, ethanol and acetic acid concentrations were measured in the growth medium (Initial) and at the end of fermentations (Final). Three biological triplicates were performed for the three tested conditions, error represents the standard deviation between replicates. [file 13068_2021_1880_MOESM2_ESM.png]

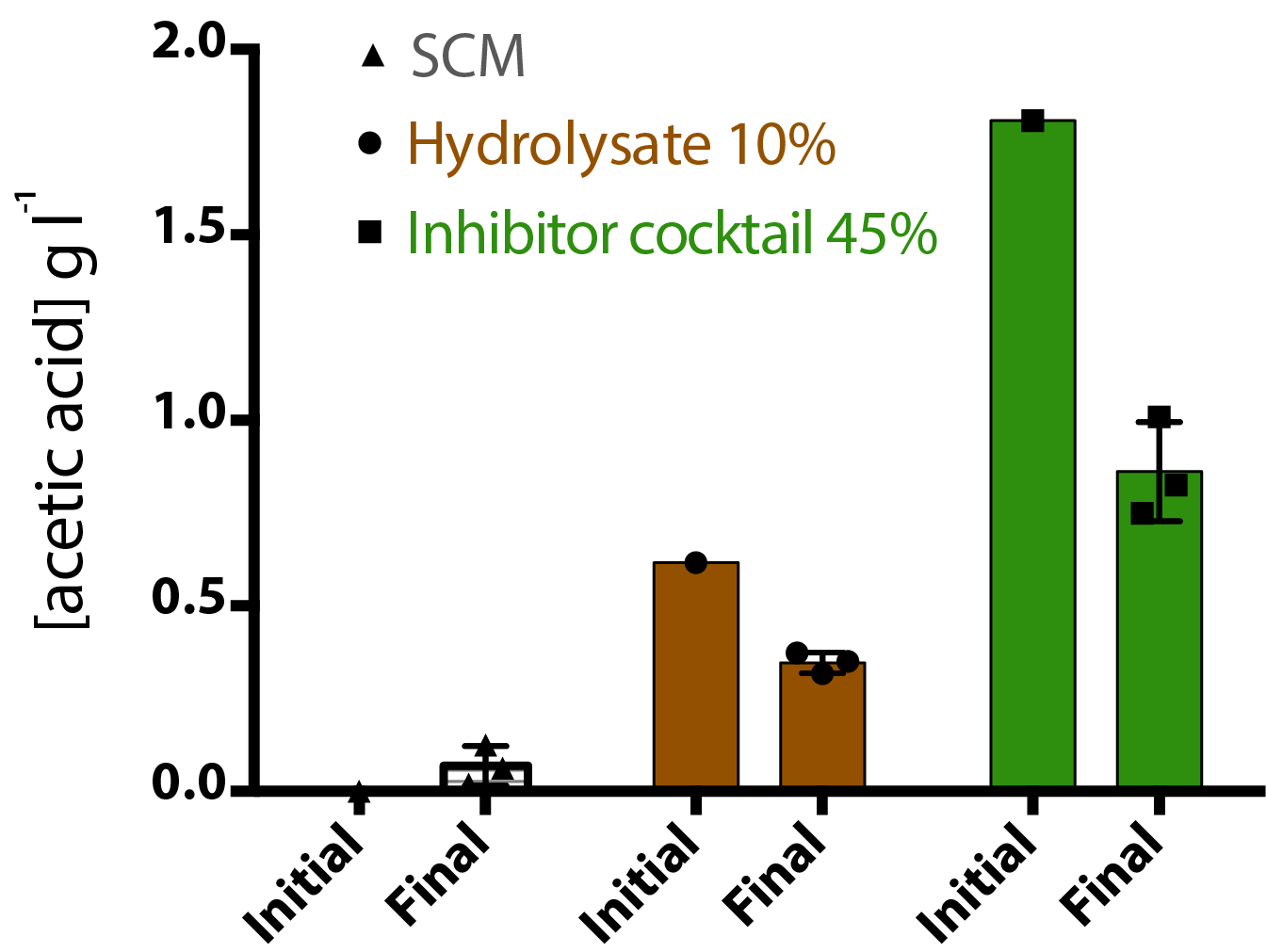

Supplement: Supplementary file 3 — Additional file 3: Figure S2. Acetic acid metabolization. Changes of acetic acid (in g L−1) concentration during fermentation in different growth conditions (indicated in figure legend) at cultivation start and end points, measured by HPLC. Initial data corresponds to media used to inoculate, while each point in “Final” correspond to acetic acid concentration of three biological replicates. [file 13068_2021_1880_MOESM3_ESM.png]

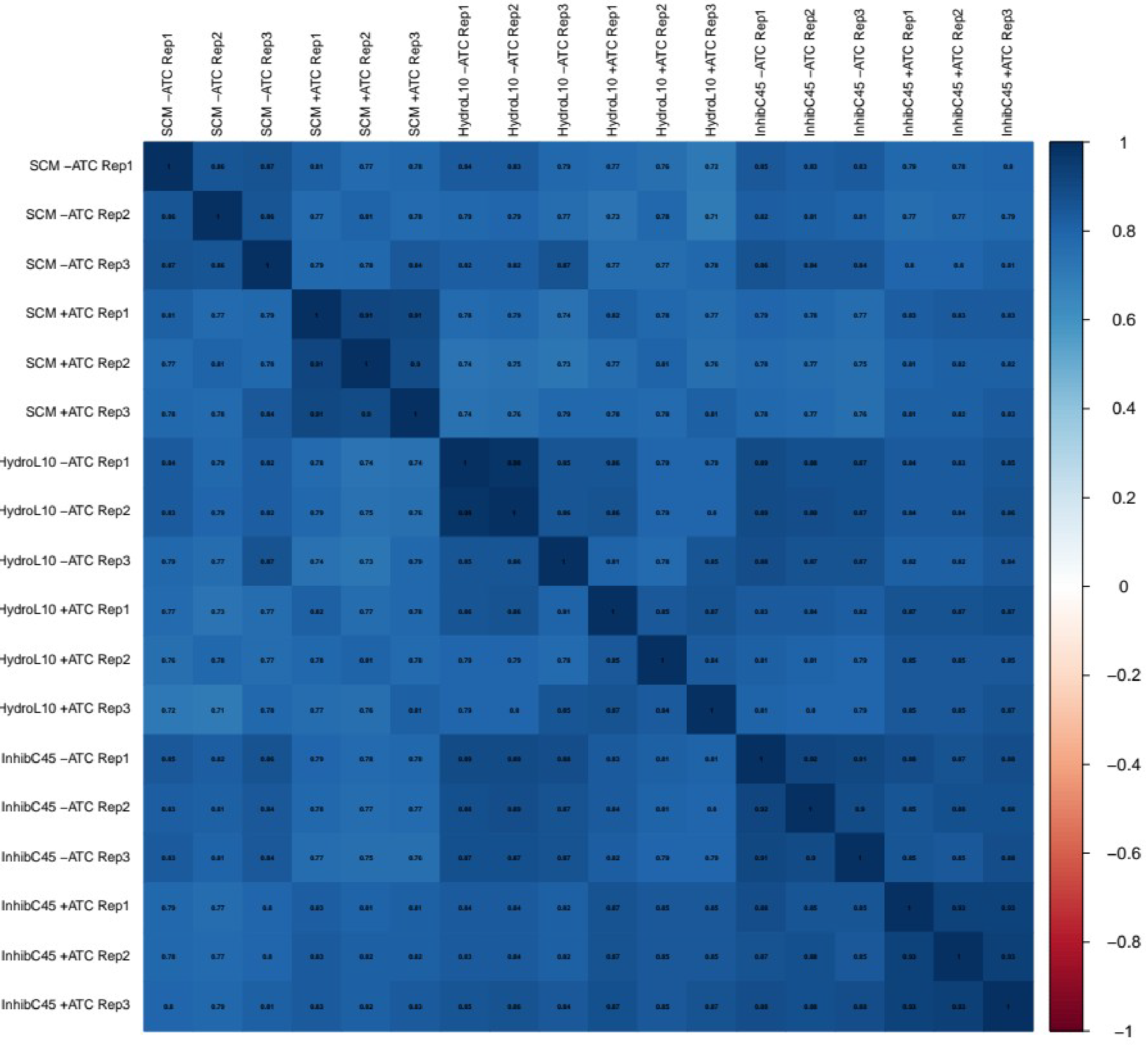

Supplement: Supplementary file 4 — Additional file 4: Figure S3. Read count correlation. Spearman correlations of read count samples across screens. [file 13068_2021_1880_MOESM4_ESM.png]

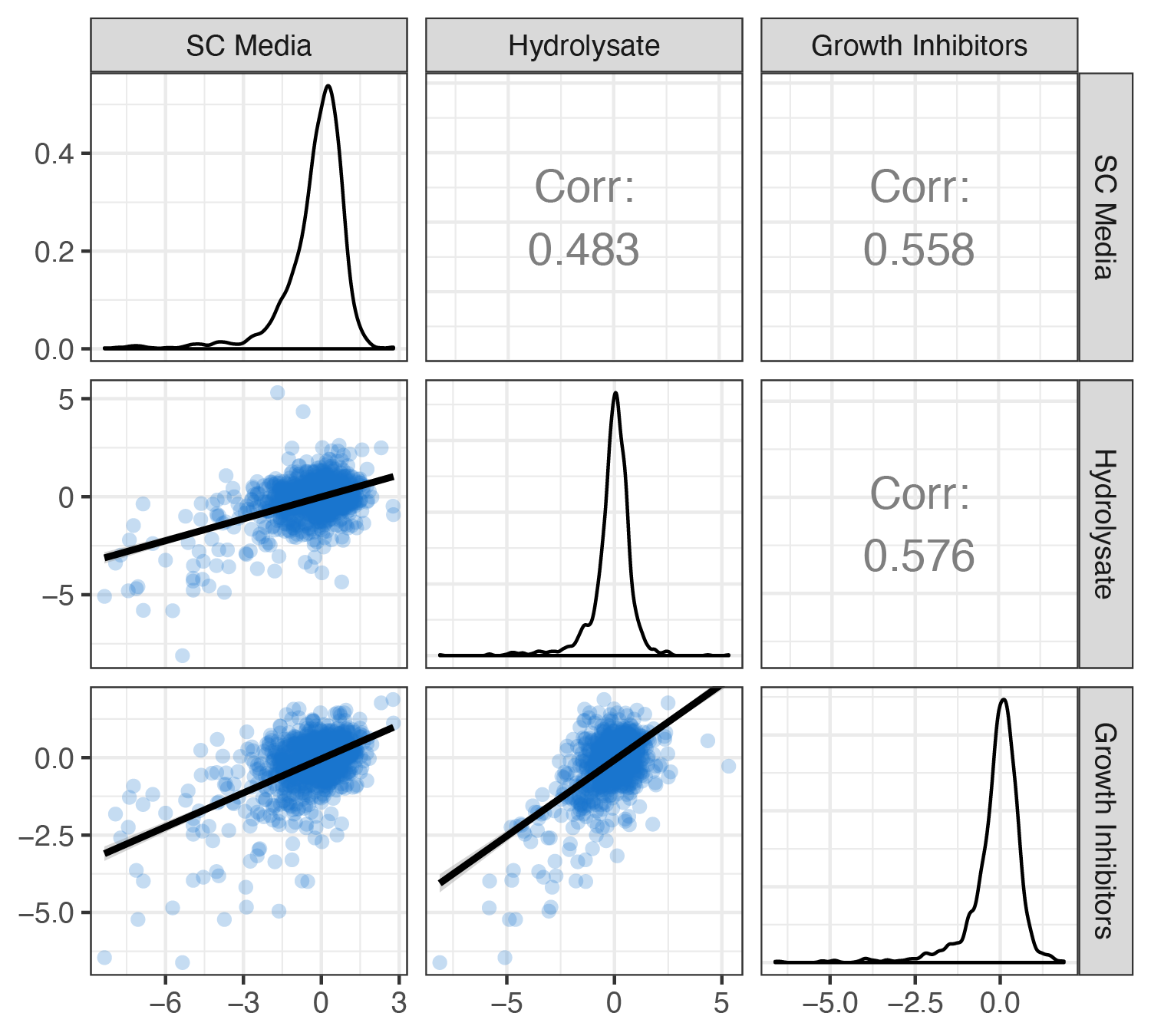

Supplement: Supplementary file 5 — Additional file 5: Figure S4. Guide RNA fold changes across conditions. Scatter plots with dots denoting gRNAs, density distributions and Pearson correlations of gRNA log2 fold changes across screen conditions. [file 13068_2021_1880_MOESM5_ESM.png]

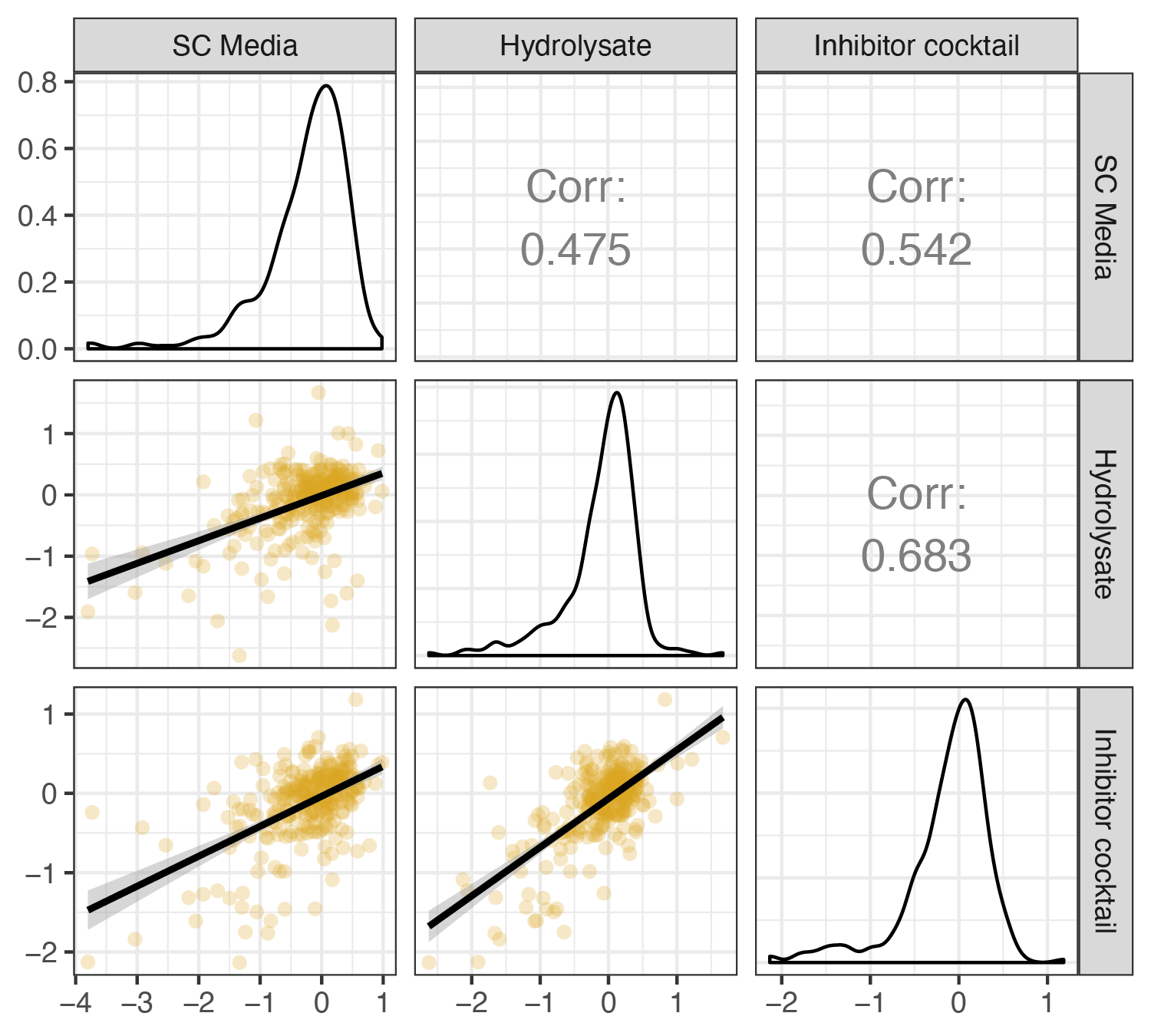

Supplement: Supplementary file 6 — Additional file 6: Figure S5. Gene fold changes across conditions. Scatter plots with dots denoting genes, density distributions and Pearson correlations of gene log2 fold changes across screen conditions. Line denotes smoothed linear fits. [file 13068_2021_1880_MOESM6_ESM.png]

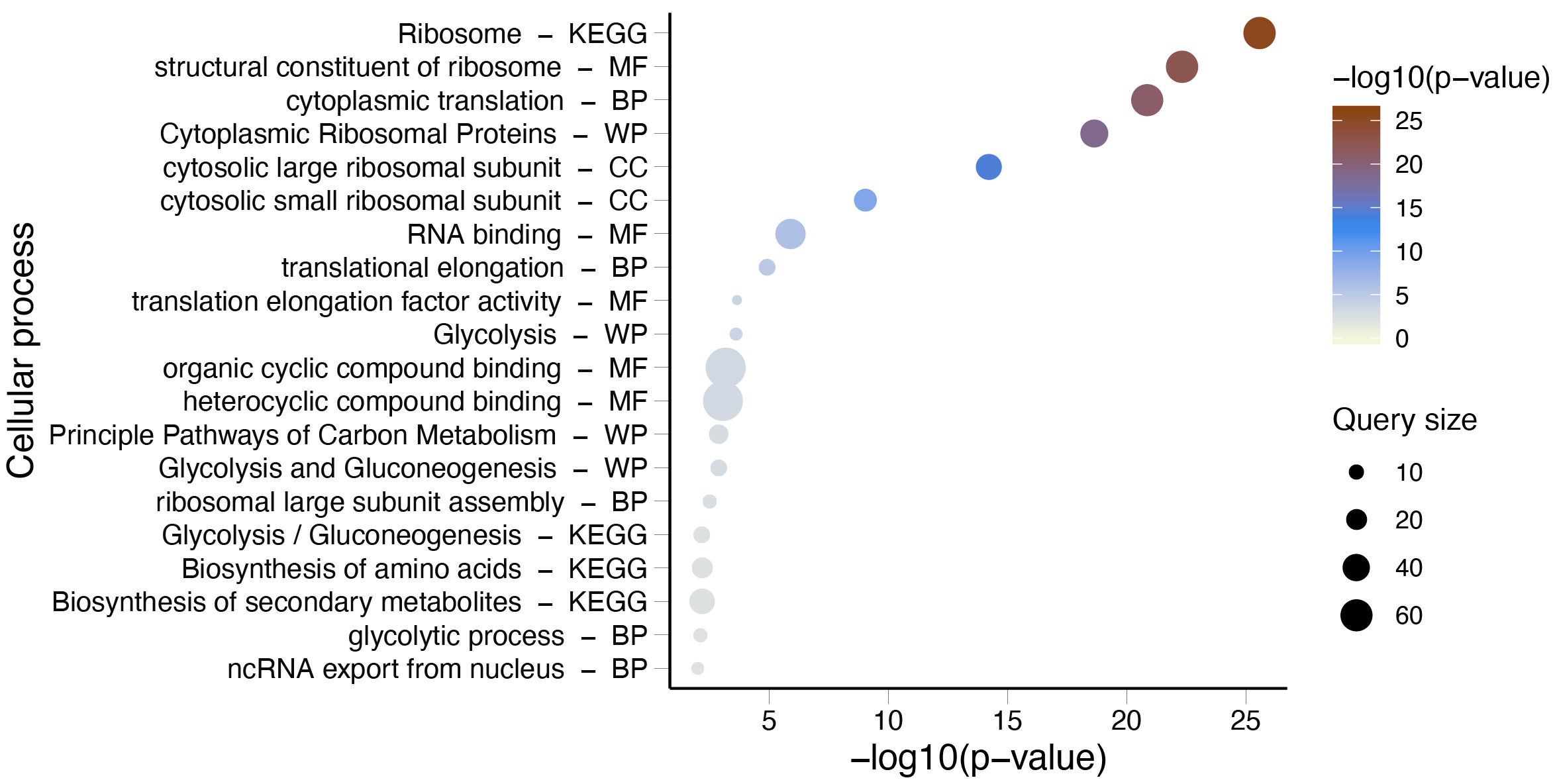

Supplement: Supplementary file 9 — Additional file 9: Figure S7. Hydrolysate-specific TF target gene functions. GO enrichment of TF target genes determined from ChIP-chip (Gonçalves et al., 2017) of TFs which modulate hydrolysate growth, generated using the gProfiler2 R package (Reimand et al. 2019). [file 13068_2021_1880_MOESM9_ESM.png]

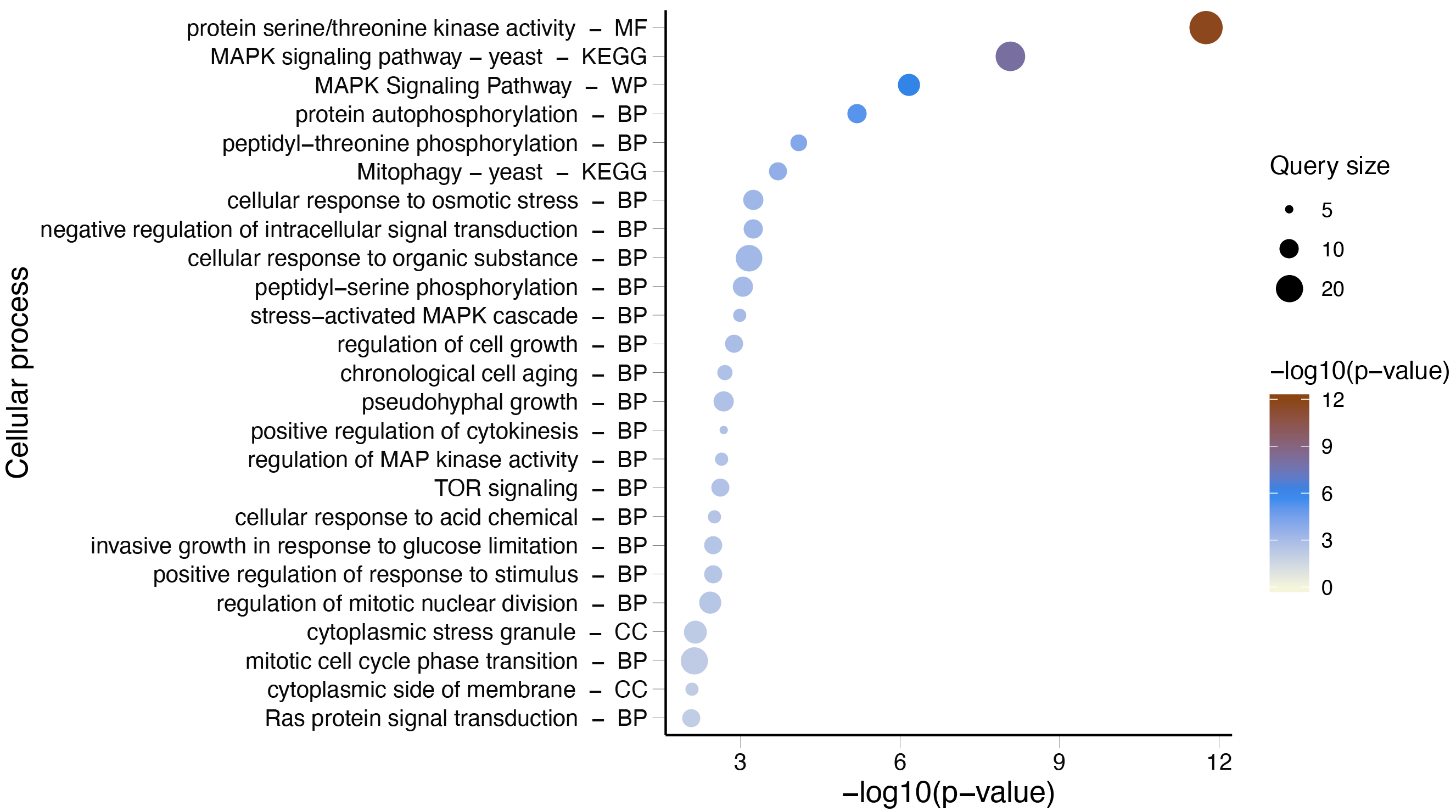

Supplement: Supplementary file 10 — Additional file 10: Figure S8. Hydrolysate-specific PK interactor functions. GO enrichment of PK phosphorylation targets determined from Phospho-proteomics data [71] of PKs which modulate hydrolysate growth, generated using the gProfiler2 R package (Reimand et al. 2019). [file 13068_2021_1880_MOESM10_ESM.png]

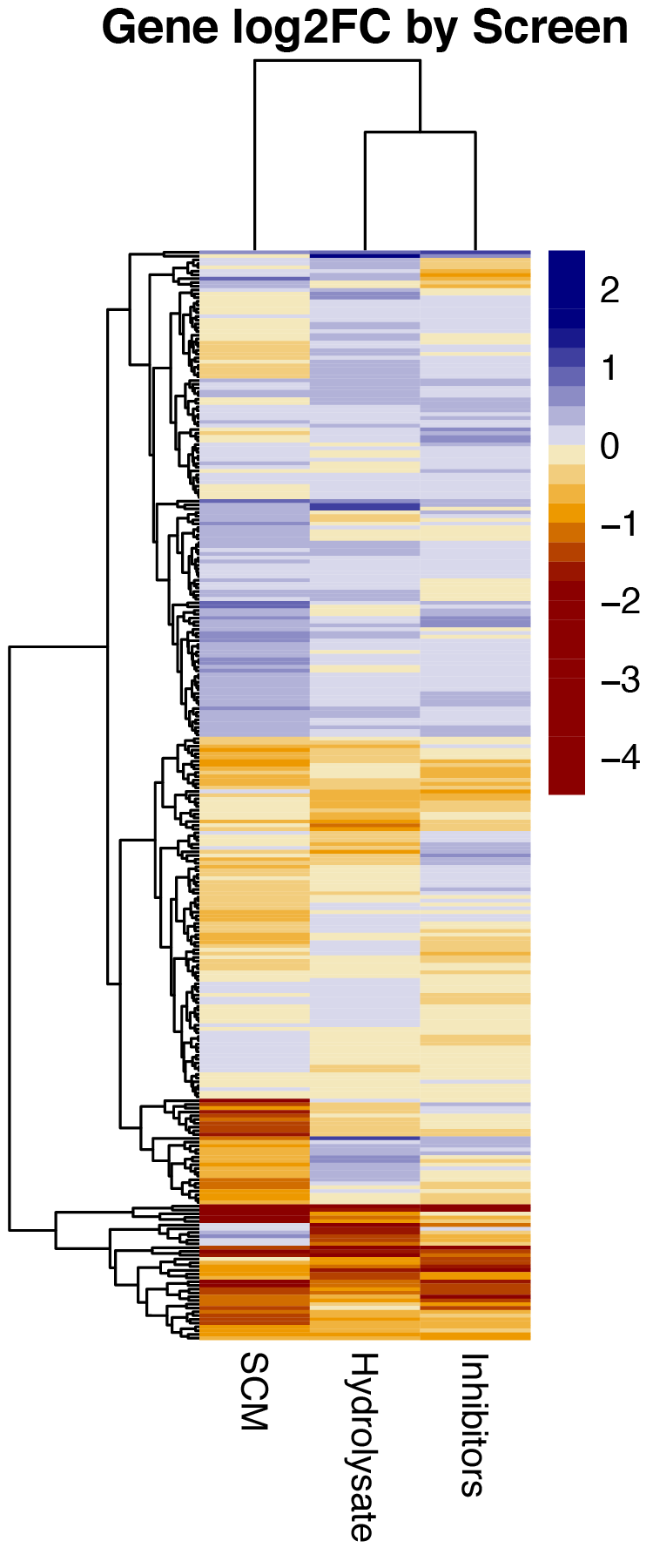

Supplement: Supplementary file 11 — Additional file 11: Figure S9. CRISPRi effects across screens. Log2 gene fold changes compared between SC medium, SCM + 10% Hydrolysate and SCM + 45% Inhibitor Cocktail. The heatmap was generated with the pheatmap R package (Kolde 2019). [file 13068_2021_1880_MOESM11_ESM.png]

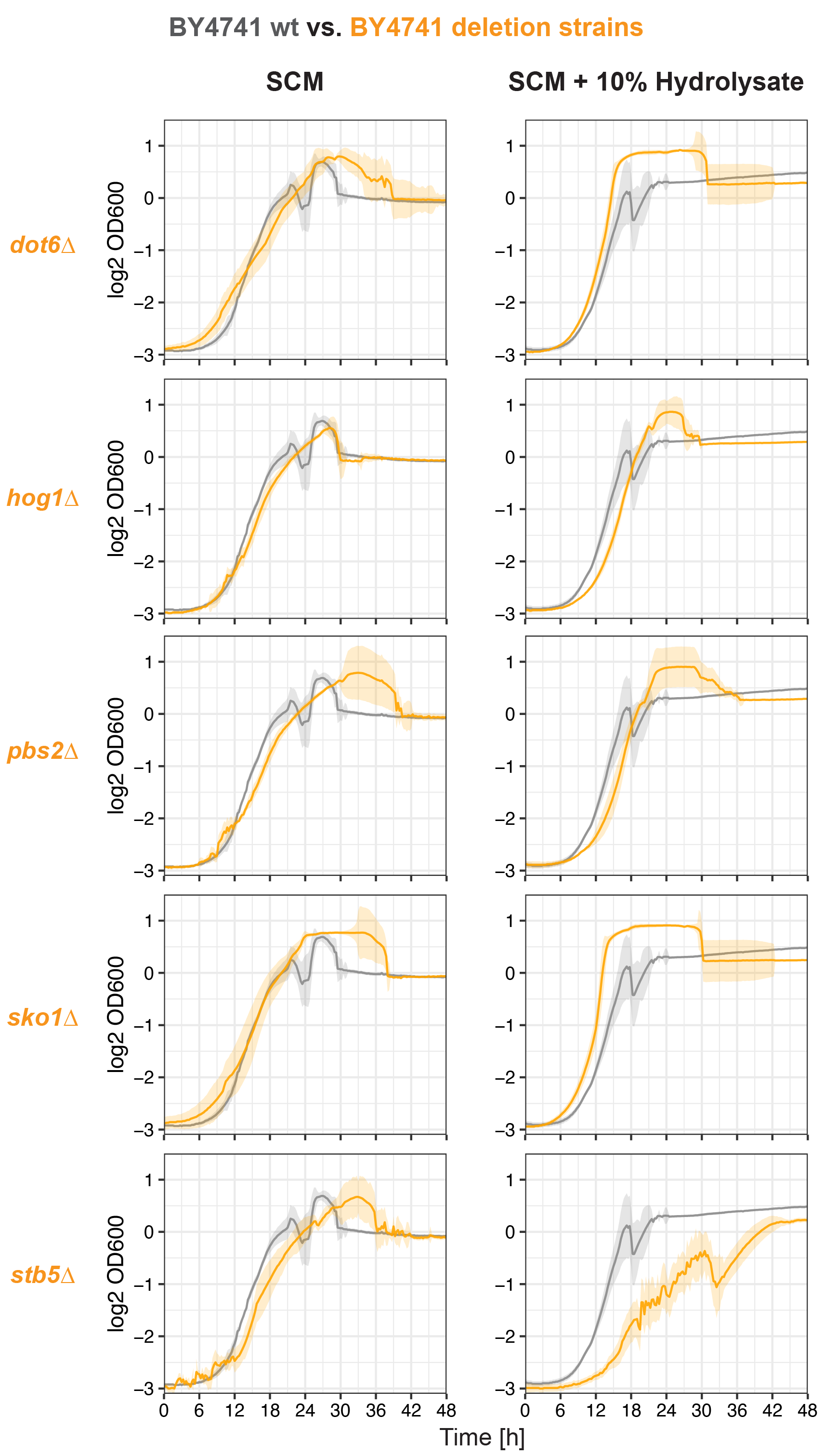

Supplement: Supplementary file 12 — Additional file 12: Figure S10. Growth profile in SCM and in SCM+10% Hydrolysate of prototrophic gene deletion strains. The optical density at 600 nm (OD600, on y-axis) was quantified over time (hour, x-axis) during growth of prototrophic BY4741 WT (grey) and the prototrophic BY4741 deletion strains (orange) in SCM and in SCM supplemented with 10% spruce hydrolysate. The curves denote the average of n = 3 wells measured in 96-well format, normalized by subtraction of media background. [file 13068_2021_1880_MOESM12_ESM.png]
